# League of Mercy

**Published:** 1905-12-23

**Authors:** 


### LEAGUE OF MERCY.

### ANNUAL MEETING OF PRESIDENTS.

Lord Mansfield presided on Friday, the 15th instant, at the 6th annual meeting of Presidents of the League of Mercy, held at the Westminster Hospital.

The following presidents, lady presidents, and hon. secretaries of districts, amongst others, were present: H.H. Princess Victoria of Schleswig-Holstein, the Duchess of Somerset, Dora Countess of Chesterfield, the Countess of Verulam, Lady Llangattock, Lady Edith Villiers, Lady Bucknill, Lady Burdett, Lady Edridge, Lady Hooker, Lady Johnson, Lady Maclean, Lady Somerset, the Hon. Mrs. Freeman-Thomas, the Hon. Mrs. Halford, Mrs. Charles Wightman, the Earl of Clarendon, the Earl of Kilmorey, the Earl of Verulam, Sir William Hart Dyke, M.P., the Dean of Carlisle, Col. Sir Fitzroy Maclean, Sir Edward D. Stern, Lieut.-Col. Sir Adelbert C. Talbot, Col. Arthur Allen Owen, Surgeon Lieut.-Col. Henry C. Thompson, M.D., Capt. G. R. B. Drummond, Capt. T. W. Gunton, Capt. Speer. The following officers were also present: Sir Henry Burdett, Sir William Collins, Mr. J. H. Harrison, Mr. E. W. Wallington, Mr. Perceval A. Nairne, and Dr. Norman Hay-Forbes.

The minutes of the last meeting having been read and confirmed, a resolution reappointing the Earl of Mansfield chairman, Mr. Frederick Green deputy-chairman, and Dr. Norman Hay-Forbes hon. secretary was agreed to, as was also one appointing seven representative members of the Executive Committee as follows: Lord Mansfield, Lord Farquhar, Mr. R. C. Antrobus, Mr. Frederick Green, Mrs. R. L. Harrison, Mrs. Buxton Morrish, and Mr. Montagu Sharpe.

Sir Henry Burdett then made a statement as to the financial results of the past year's work. He said that though it had not been a good year for raising money he thought the League was to be congratulated that on the whole they had collected a larger amount than in the year before. In 50 districts they had obtained more than £100, and these were as follows: At the head of the list was Fulham with £1,166, an increase of £98. (Applause.) But since the ideal amount to be raised by a district was £1,200 there was still room for another district to beat Fulham in the future. Then came South Kensington with £1,135, and, though less than last year, this was really a better result from the League's point of view, since it represented more work on their part and less help from extraneous sources. Next on the list was the combined district of four counties under the presidency of Her Highness Princess Victoria of Schleswig-Holstein, which totalled £1,048, being made up of Berks £668, Bucks £222, Oxon £102, and Hants £56, the last-named counties having only recently been organised. South Paddington came next with £962, an increase of £256, for which he should like to express the gratitude of the Executive to the hon. secretary of the district, Miss Wakefield. (Applause.) Next in order were St. George's, Hanover Square, £960; Sevenoaks, £608; St. Pancras North, £585; Strand, £50'; Kingston and Surbiton, £471; Epsom, £447; Chelsea, £428; Tonbridge, £364; Westminster, £356; Hampstead, £340; Marylebone West, £346; Lewisham, £340; Dulwich and Norwood, £338; Marylebone East, £324; Deptford, £292; Kensington North, £281; Richmond, £256; Chelmsford, £246; Woolwich, £237; Ealing, £222; Hammersmith, £214; Chertsey, £210; Eastbourne, £200; Epping, £200; Brentford, £188; City of London, £188; Hertford, £184; Guildford, £161; Camberwell North and Newington, £161; Tower Hamlets, Whitechapel, £157; Croydon, £155; Maldon, £145; Greenwich, £139; Reigate, £135; Isle of Thanet, £134; Lambeth (Brixton), £132; St. Pancras West, £132; Islington West, £130; Horsham, £127; Tower Hamlets, Mile End, £125; Romford, £123; Hackney North, £116; Wimbledon, £116; Colchester, £103; Medway, £101; and Essex, S.E., £100. There were then a large number of districts with smaller amounts, among which he might mention West Southwark with £40, due mainly to the energy of a foreman in the building trade who had endeavoured to interest his fellow-workmen. The total collected for the year was £17,500, an increase of about £1,000—(applause)—and the Executive had decided to increase the grant to the King's Fund from £14,000 to £15,000. The expenses had also been reduced, and though the work had been extended they had managed to save about £100, which showed that care was taken to keep the expenditure down as far as was possible. It was of great importance that they should go on making grants to the hospitals in the districts. (Applause.) There was no doubt as to the good work done by the visits made to the metropolitan hospitals by the King's Fund, and the League proposed to follow that good example and send visitors, and base the awards made on their reports as to the needs and circumstances of each institution. That, he was sure, ought to help with the work and encourage them to persevere. They knew, of course, that the success of the League depended largely on the vice-presidents, who were their recruiting sergeants, to enlist workers. Many of them had intimated that they had a feeling of isolation, and to remedy it suggested that as the Presidents had an annual meeting, why not the Vice-Presidents also? He thought that seemed a practical idea, and if the Presidents saw no objection it was hoped that it would be carried out in the coming year. If the meeting were held in a large place where they might hear addresses, he thought it might extend the interest. Also he thought that every district should have at least one hon. secretary, since continuous work was needed in looking after the recruits. He could assure them that at the central office they were very sensible of the devotion and hard work displayed by the hon. secretaries generally. He had heard of instances where some of the workers seemed to be getting a little tired. They had joined the League with energy and devotion, and in their enthusiasm had perhaps a little overdone their powers, and felt they would like to take up with something rather less onerous and difficult. He hoped that every President and Vice-President would give their measure of personal service in seeing every tired worker and encouraging each to persevere with the desire that should animate all in the days of health to help the cause of the sick and suffering poor, so that they would come to feel that they could not find it in their hearts to turn back from the good work to which they had put their hands. He asked them to take to all who seemed to flag the sentiment which he had brought back from the address of an American professor to his medical students: "The poor—they cannot pay; but thou shalt be paid." (Applause.)

Lady Llangattock and Mr. Carl Hentschell made brief statements regarding the work in their respective districts, after which

Sir William Collins said that although there was nothing sensational to report during the year, the League grew steadily, and substantial progress had been made. New districts had been opened and old ones advanced. Among the new presidents added during the year were the Duke of Bedford; and in July the Prince of Wales had again entertained between 900 and 1,000 workers of the League at a garden party at Marlborough House, when 55 Orders of

Mercy were conferred. It was certainly an occasion of gratification to see the army of the League drawn out in long array. A matinée concert had been held at Grosvenor House, when a profit of £374 was realised; and only that week a successful dance had taken place at the Empress Rooms, Kensington, from which it was anticipated that from £180 to £200 would be received. The League indeed continued to be a very valuable ally of the King's Fund, and the £15,000 they were handing over this year made a total of £61,000 contributed to that fund since the League was constituted. That represented nearly one-sixth of the total collected by the fund, as compared with exactly one-seventh last year. The League made no sensational appeal, but contented itself with a plain statement of the facts; while every shilling collected went direct for the purpose for which it was contributed.

Mr. Cecil Fane de Salis, President of the Uxbridge district, suggested that collecting boxes might be employed, and Sir William Collins said the proposal would certainly be considered at the next meeting of the Executive.

Lord Kilmorey moved a vote of thanks to the authorities of the Westminster Hospital for the use of the board-room for the meeting, and a vote of thanks to the Chairman closed the proceedings.

